# Chronic pain in families: a cross-sectional study of shared social, behavioural, and environmental influences

**DOI:** 10.1097/j.pain.0000000000001062

**Published:** 2017-09-11

**Authors:** Paul Campbell, Kelvin P. Jordan, Blair H. Smith, Generation Scotland, Kate M. Dunn

**Affiliations:** a Arthritis Research UK Primary Care Centre, Institute for Primary Care and Health Sciences, Keele University, Keele, Staffordshire, United Kingdom; b Division of Population Health Sciences, School of Medicine, University of Dundee, Dundee, United Kingdom; c Generation Scotland, Centre for Genomics and Experimental Medicine, Institute of Genetics & Molecular Medicine, University of Edinburgh, Edinburgh, United Kingdom

**Keywords:** Chronic pain, Family, Multi-level modelling, Social, Generation Scotland, Concordance

## Abstract

Chronic pain is common and creates a significant burden to the individual and society. Emerging research has shown the influence of the family environment on pain outcomes. However, it is not clear what shared factors between family members associate with chronic pain. This study aimed to investigate the family-level contribution to an individual's chronic pain status. This was a cross-sectional study using the Generation Scotland: Scottish Family Health Study data set. This study focused on a nested cohort of dyads (only 2 relatives per family, n = 2714). Multi-level modelling was first performed to estimate the extent of variance in chronic pain at the family level. Then each member of the dyad was randomly assigned as either the exposure or outcome family member, and logistic regression was used to identify shared factors associated with the outcome of chronic pain status. Multi-level modelling showed just under 10% of variation in chronic pain status was at a family level. There was an increase in odds of chronic pain if exposure family member had chronic pain (odds ratio [OR]: 1.30, 95% confidence interval [CI]: 1.02-1.65), if both were women (OR: 1.39, 95% CI: 0.99-1.94), if both were older in age (OR: 1.80, 95% CI: 1.31-2.48), and if both had low household income (OR: 3.27, 95% CI: 1.72-6.21). These findings show that most explanation for chronic pain is still at the individual level. However, some significant shared effects between family members associate with chronic pain, and this highlights the influence of the family context.

## Abstract 2

Little is known about the family-level influence of chronic pain. Our findings show a contribution to chronic pain status from shared family characteristics.

## 1. Introduction

Chronic pain is common within the population and has an impact on the individual, their family, and wider society.^3,49^ There are complex interactions between the individual with chronic pain and their family environment. Evidence shows the influence and impact of chronic pain on family members, in terms of the adjustments family members make (for example, possible employment changes), relationship changes (for example, marital quality), and potential role changes (for example, becoming a caregiver for the person with pain and associated disability).^24,25,28,44^ The converse is also possible that the family has an influence on the individual with chronic pain; numerous studies show the effects of family members, particularly partners, on the outcomes of those with chronic pain conditions, for example, solicitous responses (eg, being overly helpful with tasks and duties), mood influences, and negative reactions (eg, anger and frustration in partners) affecting relationship quality.^6,8,9,48^ Evidence also exists of more direct influences and interactions at a biological and genetic level between family members. A number of twin and family studies have reported shared biological heritability concordance (shared risk) between family members for pain conditions.^20,22,23,47^ For example, Hocking et al.^22^ report that the genetic heritability estimate for chronic pain was 29% in a study of 2195 extended families, and another study^20^ has shown a significant association between maternal and related adolescent chronic pain.

Research on specific conditions such as face pain, stomach pain, and headache has shown that family members are more likely to have similar symptoms, or have elevated levels of poor health compared to nonfamily members.^11,27^ Families are also likely to share similar lifestyles, and express similar health behaviours and beliefs,^18,29^ and a significant amount of health care engagement can be explained at a family level.^12,13^ Furthermore, families are likely to share the same environment, and so share similar economic status, educational status, and access to health services.^10,34,39^ Recently, an article described the concordance between partners (eg, husband and wife) for musculoskeletal pain; concordance was partly explained in terms of the shared lifestyle and environment between couples.^7^ Overall, this evidence suggests that, aside from biological and genetic propensity, there might be other important shared influences to explain concordance between family members. A recent heritability twin study conducted by Vehof et al.^47^ shows that 7% to 10% of the variance in chronic pain syndrome is explained by the common environment (ie, shared social factors) over and above genetic and individual contributions. Clearly shared effects between family members are present, but currently we do not know what the specific shared factors are that may result in increased concordance for pain conditions. The aim of this study was to investigate the family-level contribution to chronic pain status within the individual, and describe which shared factors are associated with chronic pain.

## 2. Methods

### 2.1. Design and participants

This is a cross-sectional analysis of participants in the Generation Scotland: Scottish Family Health Survey (GS:SFHS^41^). Briefly the GS:SFHS identified potential participants at random from people aged 35 to 65 years registered at collaborating primary care medical practices throughout areas of Scotland. Participants were invited to take part and to identify at least 1 first-degree relative (ie, the index person's mother, father, sister, brother, and adult child) aged 18 years or older to also take part. Volunteers from anywhere in Scotland were also welcomed to participate in GS:SFHS, again with the request that one or more first-degree relatives (aged 18 years or older) also agree to take part. In total, 126,000 probands were invited with 12.3% volunteering and meeting the Generation Scotland inclusion criteria.^42^

Participants completed preclinic health questionnaires and attended research clinics for a physical examination, and mental health and cognitive function assessments. In total, at the time of this study, 21,327 individuals were participating forming 2195 family groups. Fuller details of the recruitment process are given elsewhere (Ref. 41,42, www.generationscotland.org). The GS: SFHS was approved by the Tayside Committee on Medical Research Ethics (reference 05/S1401/89).

The current study focuses on a nested cohort of the total population. We included index participants who only recruited 1 other first-degree relative (n = 2714 individuals forming 1357 family dyads). This strategy was specifically chosen on the basis of the analysis design, where each member of the family dyad was randomly assigned as either the exposure or outcome. This ensured that each family member was a first-degree relative, with the rationale that first-degree relatives (eg, mother, father, brother, sister, and adult child) would be more likely to experience, or have experienced, shared factors (eg, economic, physical activity, health behaviour, and psychological) compared to second-degree or more distant relatives. For example, first-degree relatives would most likely live or have lived in the same household as each other at some point, and have demonstrated continued contact with each other.

### 2.2. Measures

The outcome measure of chronic pain is based on the definition developed for the (IASP).^33^ Chronic pain was assessed within the preclinic questionnaire, and participants were asked if they currently experienced continuous or intermittent pain, and if yes, whether this pain had lasted for at least 3 months or more. Those answering yes to both of these questions were classified as having chronic pain.

Potential shared physical factors included age (categorised in age bands 18-29, 30-49, 50-69, 70+ years), sex, weight (categorised as underweight/normal vs overweight/obese/severely obese using body mass index cut-off ≥ 25). Potential shared health behavioural factors included smoking status (never smoked vs current smoker/previously smoked), and whether the participant lived with someone who currently smokes. Education level was based on the number of years the participant was at school full time or in further study full time. Three categories were created to follow the UK's Educational system (United Kingdom Government^14^), compulsory education (eg, primary/secondary education up to 11 years of education), further education (eg, college education, 12-15 years), and higher education (eg, university, >15 years). Social environment measured whether the participant lived with a partner (eg, husband, wife, or cohabitee). Financial status was measured as annual household income (categorised as £0 < £30,000, £30,000-£50,000, and >£50,000), and accommodation status categorised as own home outright, current mortgage, currently rent, and other. Finally, we measured potential shared psychological status using the general health questionnaire version 28 (categorised using the recommended cut-off score of 5 or above to indicate psychological morbidity^30,31,38^).

### 2.3. Statistical analysis

Analysis was conducted within the GS:SFHS data set. The aim of this study did not overlap with any previous study using this data. A 2-stage process was applied to address the research aim. The first stage investigated explanatory variables associated with the outcome of chronic pain across the cohort, with a multi-level model producing an estimation of the amount of variance in chronic pain status that was at the family level rather than the individual level. A 2-level hierarchical model was used, with individual participants (level 1) nested within their respective family dyads (level 2). An initial variance components model (ie, no explanatory variables entered) was performed to establish whether there was a significant effect at level 2 (family effect) using the likelihood ratio (LR) test.^43^ A variance partition coefficient (VPC) was calculated (VPC = 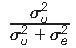 where 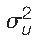 = residual variance (level 2), and 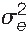 = 3.29 (logit), to estimate the proportion (%) of variance in chronic pain at the family level.^1,43^ The use of a logit function is appropriate for a binary outcome multi-level model. The standard logistic distribution (π^2^/3 = 3.29) is taken as the measure of level 1 variance, allowing for comparison on the same scale for level 2 variance, with VPC as the calculation of the ratio of level 2 variance to the sum of the level 1 and level 2 variances.^42^ Then, explanatory variables (individual's age, sex, weight, smoking status, living with smoker, education level, living with partner, household income, accommodation status, and psychological status) were then entered into the model singularly (univariable multi-level logistic regression models) to estimate the significant factors associated with chronic pain. All variables were then placed within a final multi-variable multi-level logistic regression model. This model was used to test the associations of the variables with chronic pain across the cohort (ie, the general effect of variables on outcome) with a further VPC calculation performed to produce an estimate of unexplained variance residing at level 2 (family) within the final multi-variable model (ie, proportion of variance in chronic pain status at a family level).

The second stage of the analysis considered how the significant explanatory variables from the first stage interrelate between family members to estimate the shared effect on chronic pain status. To model this, each participant within each family dyad was randomly assigned to be either an “index” family member (outcome being chronic pain status) or “exposure” family member following previous methodology.^7^ Variables significant from the multi-variable multi-level model at the first stage were then entered as shared (ie, using measures from both family members) potential predictors of chronic pain in the index participant using logistic regression producing odds ratios (ORs) and 95% confidence intervals. Statistical adjustment was made for the age of both family members and the exposure family member's chronic pain status (to ensure that shared effect was not an artefact of pain status). Using sex in association with the index family member's chronic pain outcome as an example, the analysis considered the independent association of the index family member's sex, then the independent association of the exposure family member's sex, and finally a shared analysis (ie, index family member female and exposure family member male, index family member male and exposure family member female, both family members females compared with where both family members are males). Although the use of logistic regression is appropriate for this cross-sectional design, there are issues in the interpretation of effect size (relative effect) where the prevalence of the outcome is large. It is shown for example, that the interpretation of ORs generated from populations where the prevalence of the outcome is low (ie, rare disease assumption) are comparable to estimates of relative risk; however, where prevalence of the outcome is high (eg, >10%), the reported ORs can overestimate the relative effect.^16,40^ Given that previous studies within the Generation Scotland population^22,42^ have reported a high prevalence of chronic pain status (>30%), this study will, alongside ORs, also report the prevalence percentage difference. The prevalence percentage difference will be calculated to show the difference from the reference category prevalence of chronic pain and the influence of exposure from both the index family member and the exposure family member. Complete case analysis was performed because of the low level of missing data,^41^ and analysis was performed using SPSS version 21 and STATA 13 (STATA binary level multi-level modelling command *xtmelogit*).

To determine whether the 2 member family dyads in this current study were different from those within Generation Scotland with more family members (eg, 3, 4, 5, 6, 7 or more), we compared the size of family block across a range of variables (chronic pain status, age, sex, body mass index , smoking status, education level, living with a partner, household income, accommodation status, and psychological morbidity) using 1-way analysis of variance (continuous variables) or χ^2^ (categorical) tests. These tests show no significant differences on any variable dependent on family block size (data not shown).

## 3. Results

Characteristics of the cohort are described in Table 1. The mean age was 47 years (SD 15 years), 59% were women and just over 36% of the cohort indicated the presence of chronic pain.

**Table 1:**
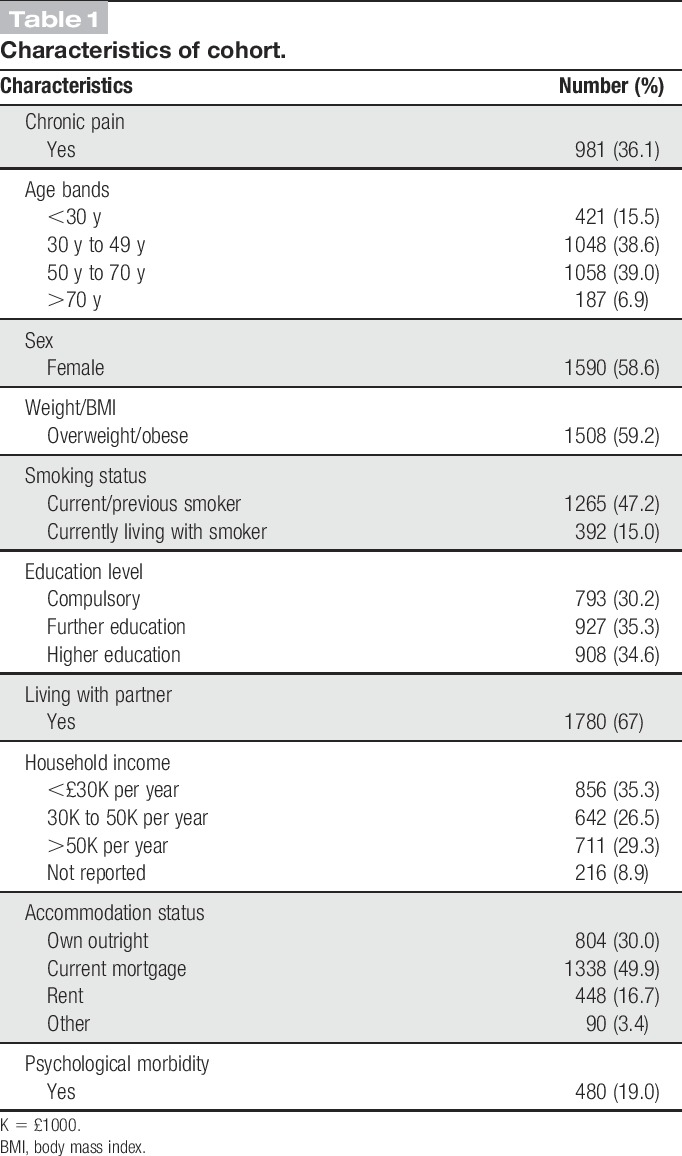
Characteristics of cohort.

### 3.1. Stage 1: multi-level modelling

Table 2 shows the results of the multi-level logistic regression analysis (stage 1). The multi-level univariable logistic regression results showed that age (being in older age bands), sex (being female), smoking status (currently or previously a smoker), living with a current smoker, educational level (having fewer years of education), household income (having less income) were all associated with increased odds of chronic pain. Having a mortgage (compared with owning your home outright) decreased the odds of reporting chronic pain. Being overweight or obese, not living with a partner, and having psychological morbidity were not significantly associated with chronic pain. The final multi-level multi-variable logistic regression model showed that female sex, increased age, lower income, and smoking were significantly associated with increased odds of reporting chronic pain. The initial multi-level variance components model (ie, no explanatory variables added) indicated a significant family-level effect (LR test 4.81, *P* = 0.01) with 8.1% of variation in chronic pain status residing at the family rather than the individual level. Likelihood ratio tests for all univariable and multi-variable models were significant, indicating the presence of a significant family-level effect, and the final multi-level multi-variable model VPC was 9.8% (LR test 4.15, *P* = 0.02, 9.8% unexplained variance at the family level).

**Table 2:**
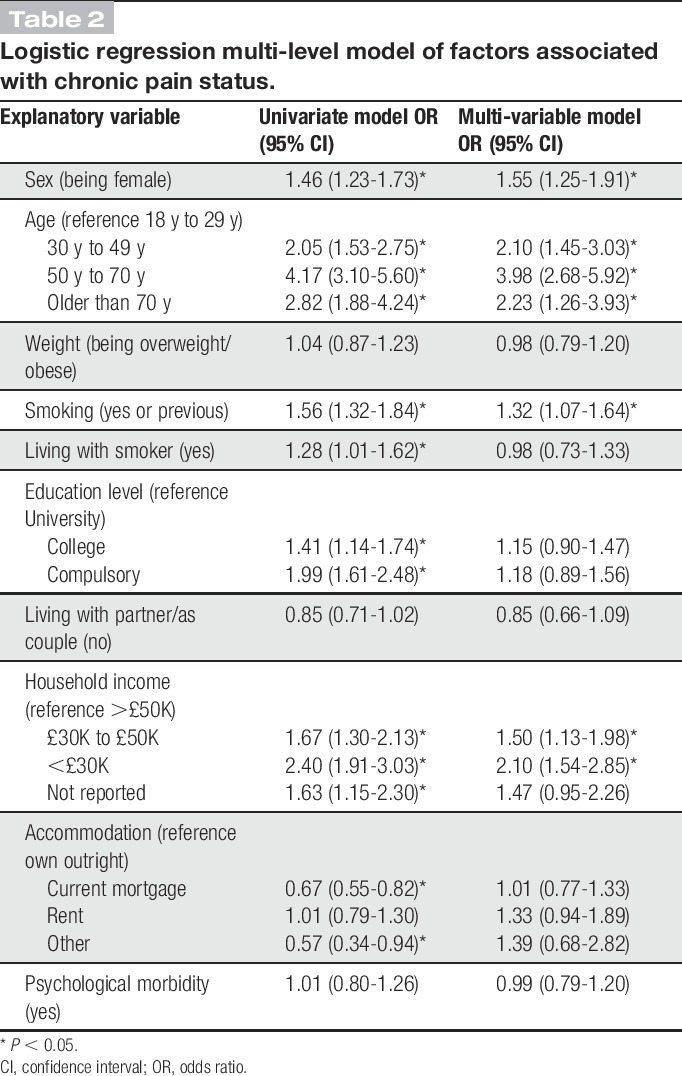
Logistic regression multi-level model of factors associated with chronic pain status.

### 3.2. Stage 2: shared effect analysis

Table 3 outlines the shared effects of the significant factors associated with chronic pain from stage 1. This shows that when the exposure family member indicates they have chronic pain, there is a 30% increase in odds of reported chronic pain in the index family member (after adjustment for both index and exposure family members' age), prevalence percentage shows an increase of 5.9% addition due to the exposure having chronic pain. The effects of sex show, using both family members as male as the reference category, that being female (index family member) gives a prevalence percentage increase of 4.5%, but if the exposure family member is a female (and index male) there is a reduction (−0.7%); both results were not significant within the logistic regression tests. However, when both family members are females, independent of the exposure family member's chronic pain status, there was a nonsignificant trend (adjusted OR: 1.39; 95% confidence interval: 0.99-1.94) with a prevalence percentage increase of 9.1%, which is a 4.6% increase on the effect if the index family member is a female. Considering the shared effect of age, compared to when both family members are within the youngest categories (<50 years), there was a significant effect when the index was older with a 16.9% increase in prevalence, but a nonsignificant effect when the exposure was older (3.0% increase in prevalence). There is a significant effect when both index and exposure were older; the percentage prevalence increase was 14.3%, which is a reduction of 2.6% prevalence compared with when only the index was older. For income, there is a significant effect when the index person is within the low-income category, regardless of the exposure family member's income status. However, the strength of effect is stronger when both exposure and index are low income (OR: 3.27, prevalence increase of 28.2%) compared with when the index is low income and the exposure is either medium income (OR: 2.88, prevalence increase 25.5%) or high income (OR: 2.84, prevalence increase 24.3%). There is also a significant effect when both the index and exposure are within the medium-income category (OR: 2.45, prevalence increase 18.6%), and this effect is stronger when the index is within the medium-income category and the exposure is within the low-income category (OR: 2.80, prevalence increase 22.1%). Smoking only showed a significant effect if the index family member smoked or smokes (OR: 1.41, prevalence increase of 9.3%), with no significant effect found when both family members smoke or have smoked, compared with when they both have never smoked.

**Table 3:**
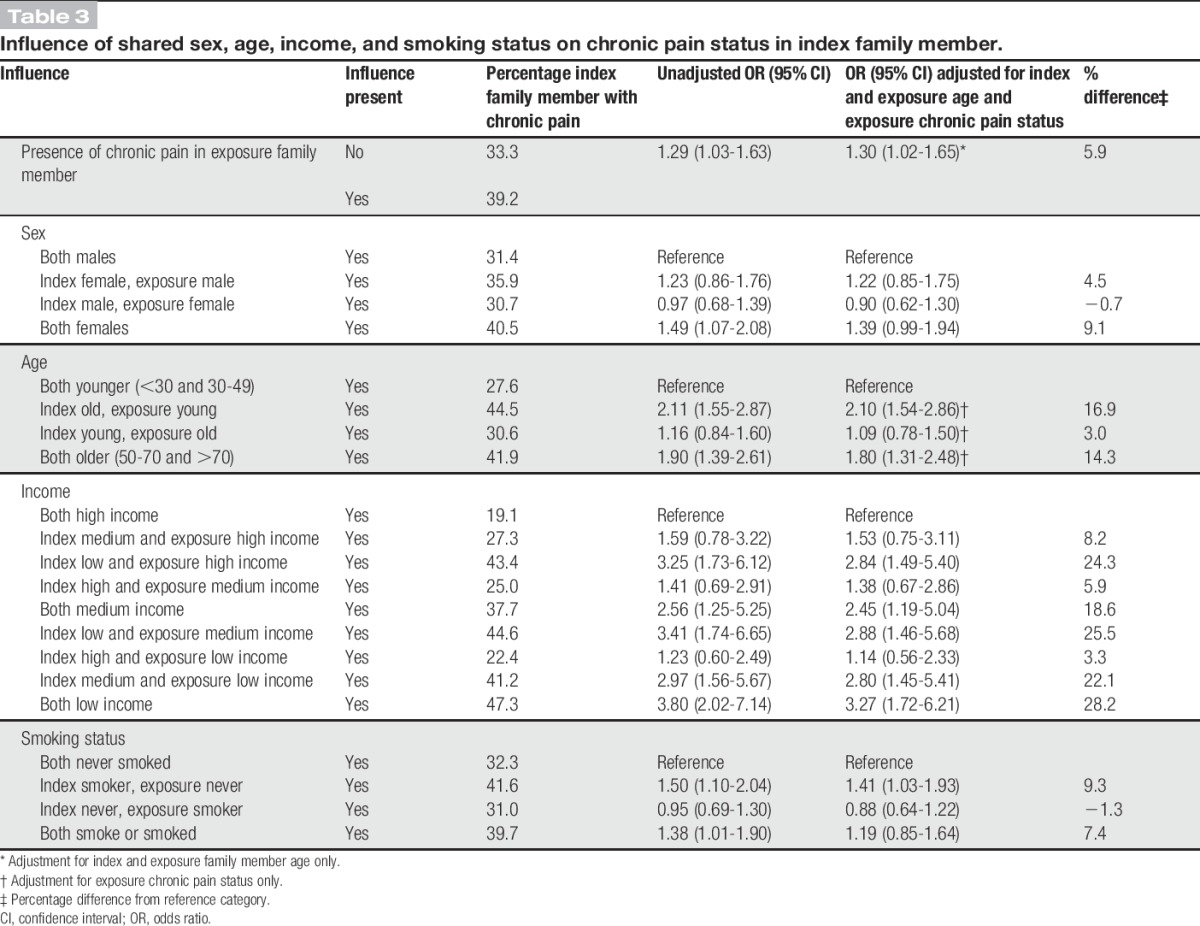
Influence of shared sex, age, income, and smoking status on chronic pain status in index family member.

## 4. Discussion

This multi-level modelling study shows that 8% of the variance in chronic pain status within a family health survey can be explained at a family level, and this rate increased slightly to 9.8% when introducing individual-level variables associated with chronic pain. Overall, this suggests that factors related to chronic pain status are mostly explained at the individual level, but that there is a modest level of shared effect present. The results of tests between family members on variables associated with chronic pain do show some effects; family members have increased odds of reporting chronic pain if they have another family member who also has chronic pain. Additional shared factors between family members that may contribute to chronic pain status were also identified, such as the shared sex status between family members, and also shared income status between family members. These findings show some potential shared effects beyond the individual that can contribute to chronic pain.

### 4.1. Comparison with other literature

In terms of generalisability, the GS:SFHS has been compared with the Scottish general population,^41,42^ and it is reported that GS:SFHS participants are generally older, but have a lower prevalence of general illness, with lower levels of chronic pain status (32% vs 46%), less likely to smoke, more likely to have a better level of education, and less likely to be depressed. Similar trends are found in the nested cohort in this current study. A recent study using the GS:SFHS data set that examined genetic heritability variance for chronic pain status report that 8% of the variance for chronic pain was explained by unmeasured “shared” environmental factors.^22^ Similarly, Vehof et al.^47^ found that a range of 7% to 10% of the variance of chronic pain syndrome was explained by common shared environmental factors, and both these figures are similar to the variances reported within this current study. We have now added to this literature by investigating what shared factors contribute to this shared effect, and the size of the effect for each variable. This current study is also in accord with other chronic pain studies in identifying, age, sex, income, smoking status, and education level as factors associated with chronic pain.^19,21,32,36,46^ Although the results report on significant shared effects in accord with previous literature, the actual contribution above and beyond the individual effects (ie, the added effect) is small. For example, the percentage prevalence of chronic pain status increased by only 5.9% if the exposure family member has chronic pain. The results for age actually show a reduction in the increase of prevalence when both family members are old (14.9%) compared with when the index family member is old (16.9%). Similarly for income, although there is an increase in chronic pain prevalence (increase of 28.2%) when both family members are low income, this is largely driven by the index individual's income status, for example, we only see a 3.3% rise in prevalence if the exposure family member is low income and the index is high. Caution should be exercised on the interpretation of percentage prevalence increase in this context, as causation cannot be assumed within this cross-sectional design. This current study did not find psychological morbidity (as measured by the general health questionnaire version 28) as a factor associated with chronic pain despite other epidemiological studies finding such an association.^2,35^ This may be a reflection of the overall lower proportion of chronic pain and psychological distress within the GS:SFHS population, compared with Scottish population norms. For example, the proportion of those depressed is double within the Scottish general population (8%) compared with GS:SFHS (4%), and the proportion of those with chronic pain at a Scottish national level is reported as 46%, whereas within the GS:SFHS, it is lower at 32% for the full cohort,^42^ and 36% within this nested cohort.

### 4.2. Strengths and weaknesses

A key strength of this study is the recruitment of a random sample of families from a diverse range of areas within Scotland. Participants included within this analysis were not recruited on the basis of their chronic pain status, and so results would be less likely influenced by response bias. Furthermore, we randomised which participant was assigned as the index family member, and which family member was assigned as the exposure family member, again to minimise bias. We also choose to only include participants who had only 1 other family member within the data set. This was for the analysis model, whereby we randomly assigned each member to either exposure or outcome status with an assumption that first-degree relatives would have increased contact with each other (as evidenced by the invitation to take part in GS:SFHS from one family member to the other) as this would increase the likelihood that family members share a current relationship and probably share similar environmental influences.^11^ However, it is acknowledged that different analysis methods could have included all Generation Scotland participants.

There are some other limitations to this study. First, we have no information on the amount of time each family member spends with each other, and no information on the geographic location of each family member, and so no way of quantifying the amount of shared status between family members. We also have no information on the type of linkage between family members (ie, brothers, sisters, mothers, and fathers). This study also lacks information on the family dynamics (eg, relationship quality between family members, ethnic/cultural groups, social network, and the level of support) which may have contributed more explanation at the family level. Although this study used a valid question on chronic pain status,^33^ we did not perform analysis based on the location of the pain, the duration of pain, the severity of pain, the impact on function, how the person views their pain, how they cope with their pain, or what medication or treatment they may be receiving for their pain. All of these factors may be more influenced by shared family effect, and further research is needed to look at these specific aspects between family members. Furthermore, the effects reported for chronic pain may differ for other types of pain (eg, back pain or chronic widespread pain); recent research has shown different rates of concordance for consultations about musculoskeletal pain in couples dependent on which body region they consulted about,^7^ and further research is now required to understand the potential differences on shared influence for different pain conditions. Last, we have no information on which participant, within the family dyad, reported pain first, or how long each family member has had their chronic pain. The duration of pain is likely to be an influence in terms of a pain severity indicator, but also in terms of social learning influence (eg, parent's long-term expression of pain influencing a child's reaction and coping with pain). Further longitudinal research would be required to help establish causal linkage factors between family members.

### 4.3. Clinical relevance

The findings on family effects associated with chronic pain reported here are relatively small and unlikely to have direct clinical relevance. For example, although we present a 30% increase in odds for the influence of 1 family member's chronic pain status on another, this only translates to a modest percentage prevalence rise of 5.9%. Therefore, we believe that our findings have greatest relevance at a population level, given the very high proportion of the population who report chronic pain, for example, 36% in this nested cohort, with general population estimates higher at 45%.^4,15,42^ Buchbinder et al.^5^ demonstrated the effectiveness of a public health intervention designed to alter beliefs about back pain and report moderate success in changing back pain beliefs and pain-related behaviours (eg, disability) at a full population level. However, subsequent attempts at population change have not been as successful, partly due to heterogeneity within the population, where people differ in their motivation, ability, and opportunity to affect their outcome.^17^ Perhaps one way of addressing chronic pain in this way (ie, public health) is to target at a family level, where greater homogeneity will be found, in effect considering the “family case history.” This may entail further research to ascertain shared family factors that are predictive of pain onset, and where identified, tailor interventions to reduce such risk factors at a family level. It may also be useful to examine the relationship between family members when they have pain; there is evidence of social learning influence on pain behaviour^45^ and research has shown that interventions targeting modifiable lifestyle factors and beliefs at a family level can reduce the impact of other long-term conditions such as heart disease and diabetes.^26,37^ In addition, there may be increased benefit combining the evidence we have at the individual, genetic, and family level, and direct treatment towards those individuals where there is a high risk of poor outcome.

## 5 . Conclusion

There is an increasing research interest on shared experience and shared risk of illness within families. Studies have begun to report on genetic evidence associated with chronic pain. In this study, we compliment such research by exploring the contribution of shared environmental factors. Taken together, the evidence suggests family effects are present that impact on the individual. Further research is now required to understand the interaction of influence between family members.

## Conflict of interest statement

The authors have no conflict of interest to declare.

Generation Scotland received core support from the Chief Scientist Office of the Scottish Government Health Directorates [CZD/16/6] and the Scottish Funding Council [HR03006].

This research was supported and funded (eg, access the Generation Scotland: Scottish Family Health Study dataset) by a Wellcome Trust Fellowship Grant to K. M. Dunn [083572].
